# Stereotactic Radiosurgery With vs. Without Prior Embolization for Brain Arteriovenous Malformations: A Propensity Score Matching Analysis

**DOI:** 10.3389/fneur.2021.752164

**Published:** 2021-10-12

**Authors:** Debin Yan, Yu Chen, Zhipeng Li, Haibin Zhang, Ruinan Li, Kexin Yuan, Heze Han, Xiangyu Meng, Hengwei Jin, Dezhi Gao, Youxiang Li, Shibin Sun, Ali Liu, Xiaolin Chen, Yuanli Zhao

**Affiliations:** ^1^Department of Neurosurgery, Beijing Tiantan Hospital, Capital Medical University, Beijing, China; ^2^Stroke Center, Beijing Institute for Brain Disorders, Beijing, China; ^3^Beijing Key Laboratory of Translation Medicine for Cerebrovascular Disease, Beijing, China; ^4^Beijing Translational Engineering Enter for 3D Printer in Clinical Neuroscience, Beijing, China; ^5^Beijing Neurosurgical Institute, Capital Medical University, Beijing, China; ^6^Department of Interventional Neuroradiology, Beijing Tiantan Hospital, Capital Medical University, Beijing, China; ^7^Department of Gamma-Knife Center, Beijing Tiantan Hospital, Capital Medical University, Beijing, China; ^8^Department of Neurosurgery, Peking University International Hospital, Peking University, Beijing, China; ^9^China National Clinical Research Center for Neurological Diseases, Beijing, China

**Keywords:** brain arteriovenous malformation, partial embolization, stereotactic radiosurgery, obliteration, hemorrhage

## Abstract

**Objective:** Whether partial embolization could facilitate the post-stereotactic radiosurgery (SRS) obliteration for brain arteriovenous malformations (bAVMs) remains controversial. We performed this study to compare the outcomes of SRS with and without prior embolization for bAVMs.

**Methods:** We retrospectively reviewed the Beijing Tiantan AVMs prospective registration research database from September 2011 to October 2014. Patients were categorized into two groups, combined upfront embolization and SRS (Em+SRS group) and SRS alone (SRS group), and we performed a propensity score matching analysis based on pre-embolization baseline characteristics; the matched groups each comprised 76 patients.

**Results:** The obliteration rate was similar between SRS and Em+SRS (44.7 vs. 31.6%; OR, 1.754; 95% CI, 0.905–3.401; *p* = 0.096). However, the SRS group was superior to the Em+SRS group in terms of cumulative obliteration rate at a follow-up of 5 years (HR,1.778; 95% CI, 1.017–3.110; *p* = 0.033). The secondary outcomes, including functional state, post-SRS hemorrhage, all-cause mortality, and edema or cyst formation were similar between the matched cohorts. In the ruptured subgroup, the SRS group could achieve higher obliteration rate than Em+SRS group (56.5 vs. 31.9%; OR, 2.773; 95% CI, 1.190–6.464; *p* = 0.018). The cumulative obliteration rate at 5 years was also higher in the SRS group (64.5 vs. 41.3%; HR, 2.012; 95% CI, 1.037–3.903; *p* = 0.038), and the secondary outcomes were also similar between the matched cohorts.

**Conclusion:** Although there was no significant difference in the overall obliteration rate between the two strategies, this study suggested that pre-SRS embolization may have a negative effect on post-SRS obliteration. Furthermore, the obliteration rates of the SRS only strategy was significantly higher than that of the Em+SRS strategy in the ruptured cohort, while no such phenomenon was found in the unruptured cohort.

## Introduction

Stereotactic radiosurgery (SRS) has become one standard treatment strategy of brain arteriovenous malformation (bAVM), especially those located in deep or eloquent regions with high surgical risks, and mounting studies suggested that SRS can achieve a satisfactory obliteration rate (1). Younger age, male gender, small size, small target volume, higher radiation dose, and a lone major draining vein have been found to be associated with obliteration after SRS for bAVMs (2). Partial embolization was generally used to reduce the volume of large bAVMs to facilitate the complete obliteration after the following SRS (3), and the targeted embolization for the comorbid aneurysms and arteriovenous fistulas may be beneficial in reducing the rupture risk after SRS (4, 5). Unfortunately, many recent studies implied a negative effect of partial nidus embolization on obliteration rates after SRS (1). However, those previous studies had some non-negligible limitations, such as the combination strategy applied post-embolization characteristics in the comparison of baseline, and the combination strategy tended to be used in larger bAVMs (1), which resulted in a more severe condition in the combination group. Therefore, due to the inherent differences in baseline characteristics between the combined upfront embolization and SRS and SRS alone, the comparison was flawed and unauthentic. We performed a propensity score matching (PSM) analysis based on pre-embolization baseline characteristics to compare the outcomes of SRS with and without prior embolization for bAVMs.

## Methods

### Patients Selection

We retrospectively reviewed 793 bAVMs out of the Beijing Tiantan bAVMs prospective registration research database (NCT04572568) from September 2011 to October 2014. The inclusion criteria were as follows: (1) The last treatment was SRS, (2) patients underwent single-session SRS, and (3) patients with more than 2 years clinical and radiological follow-up. The exclusion criteria were as follows: (1) patients who have received intervention other than embolization prior to SRS, (2) patients receiving staged SRS or multiple SRS, and (3) patients missing critical baseline information. Written informed consent for collecting clinical information was obtained from each patient at admission. The study was carried out according to the Helsinki Declaration guideline.

Patients were categorized into two groups, combined upfront embolization and SRS (Em+SRS group) and SRS alone without prior embolization (SRS group).

### Study Parameters

Baseline demographic, clinical features, and imaging data were collected. The baseline clinical characteristics included age on admission, sex, onset manifestation (hemorrhage, seizure, neurofunctional deficits, and others). The hemorrhagic presentation was defined as hemorrhage that could be ascribed to AVM. In terms of morphological characteristics, deep location was defined as nidus involving basal ganglia, thalamus, or brainstem. The definition of other angioarchitecture features were consistent with the reported terminology provided by the joint committee led by the American Society of Interventional and Therapeutic Neuroradiology (6). Nidus volume was calculated by the ABC/2 method on DSA (7). The Spetzler–Martin Grading System (SM), Virginia Radiosurgery AVM Scale (VRAS), and Modified Radiosurgery-Based AVM Score (mRBAS) were used to predict the long-term neurofunctional outcomes (8–10).

Clinical follow-up was conducted at the first 3–6 months and annually after discharge by clinical visit and telephone interview, and researchers who performed clinical follow-up assessments were blinded to the treatment modalities. In terms of imaging follow-up, magnetic resonance imaging (MRI) was routinely performed semiannual for the first 2 years after SRS and annually thereafter. Confirmatory digital subtraction angiography (DSA) was recommended to patients with complete obliteration on follow-up MRI. AVM obliteration was defined as a lack of abnormal flow voids on MRI or an absence of anomalous arteriovenous shunting on DSA.

The primary outcome was defined as AVM obliteration confirmed by MRI or DSA. The secondary outcomes comprised functional status, post-SRS hemorrhage, all-cause mortality, radiation-induced changes (RIC), including edema and cyst formation. The functional status was assessed by modified Rankin Scale (mRS) score system (favorable: 0–2, poor: 3–6).

### Embolization and Radiosurgery Procedures

The intraoperative embolization strategy depends on the consensus reached by a multidisciplinary meeting composed of senior neurointerventionists and neuroradiologists. A biplane angiography system was used (Siemens, Germany or Philip, Netherland), and the endovascular embolization was performed after induction of general anesthesia. In order to reduce the lesion volume and the risk of hemorrhage, we tend to embolize the lesion supplied by the main feeding artery or target embolization of the high-risk bleeding factors, such as aneurysm, arteriovenous fistula, etc. The main polymeric embolic agent used in this series is Onyx 18 (eV3, Inc.), which contains 6% ethylene vinyl alcohol and 94% dimethyl sulfoxide.

Stereotactic planning neuroimaging results are imported into Leksell Gamma-Plan workstation (Elekta AB, Elekta Company, Stockholm, Sweden) for definition and dose planning. T1 contrast-enhancement sequence and T2 sequence on 3D stereotactic MRI were used to delineate the radiation target. Dose planning was based on the location and volume of bAVM.

### Statistical Analysis

**Figure 1:**
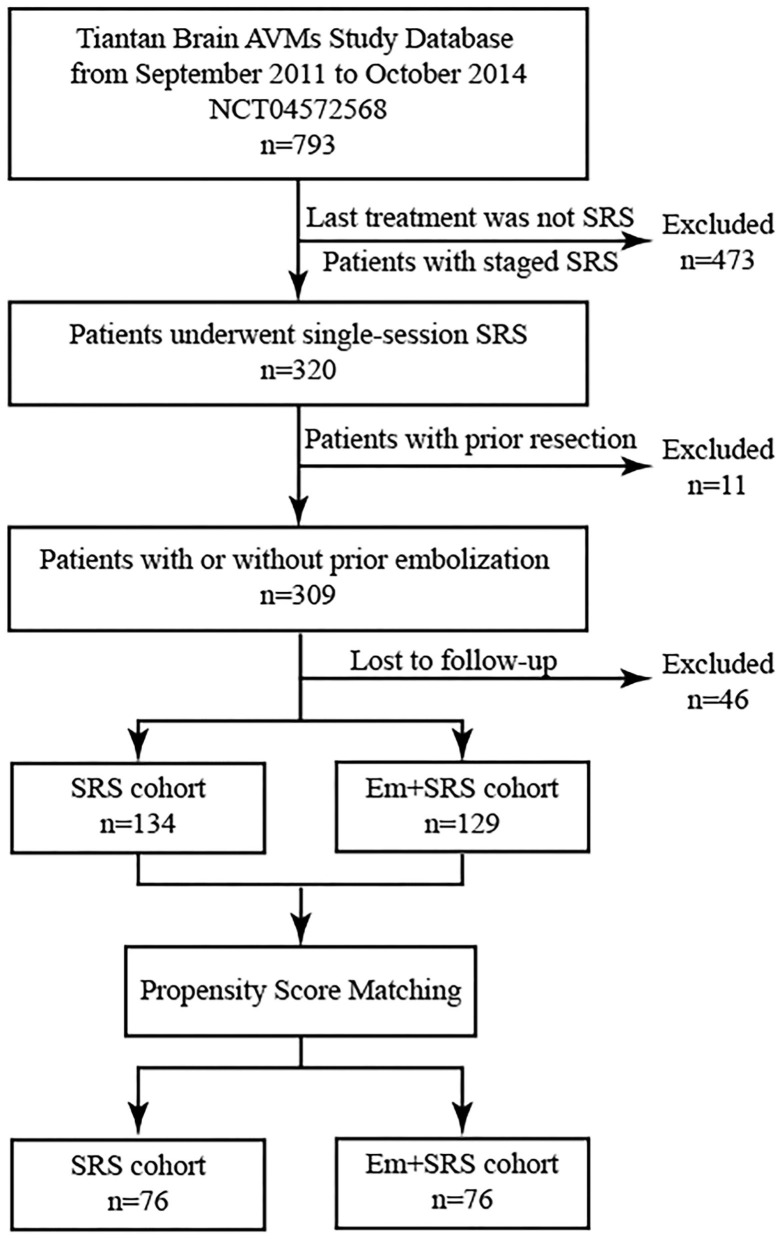
Patient flowchart demonstrating patient selection and propensity score matching (PSM) process. AVM, arteriovenous malformation; SRS, stereotactic radiosurgery; Em+SRS, prior embolization to stereotactic radiosurgery.

Categorical variables are presented as counts (with percentages); continuous variables are presented as the mean ± standard deviations (SD). A 1:1 PSM (with a caliper of 0.02 standard deviations) was performed to match the two groups with similar baseline data, such as age, gender, hemorrhage, volume, location, angioarchitecture, maximum and margin dose, and mRS at admission, mRBAS and VRAS) Absolute standardized differences was used to verify the matching results (Figure 1). At the comparison of baseline characteristics between Em+SRS and SRS, Pearson chi-square test or Fisher exact test was used to compare the categorical variables, and the two-tailed *t*-test was employed to compare the continuous variables (normal distribution variables). Wilcoxon rank-sum test was applied to compare non-normal distribution continuous variables. Univariable binary logistic regression analysis was applied to assess the odds ratios (ORs) and associated 95% confidence intervals (CI) of outcomes between these two groups. The cumulative rates of obliteration, post-SRS hemorrhage, and all-cause mobility were compared between the two groups using Kaplan–Meier survival analysis (log-rank test) to assess the hazard ratios (HRs) and associated 95% CI. Considering the difference in follow-up time and the cumulative effect of time between the two groups, we used the 5 years and the last follow-up two times nodes to calculate the *p*-value in the above survival analysis. A value of *p* < 0.05 was considered to be statistically significant. Statistical analysis was performed using SPSS (version 25.0, IBM, New York, USA).

## Results

### Baseline Characteristics

A total of 152 patients were included in this study after PSM analysis (Table 1). The mean age was 29.8 ± 13.5 years old, and 93 (61.2%) patients presented with hemorrhage. One hundred thirty-two (86.8%) lesions were supratentorial, and 35 (23.0%) niduses were classified as deep locations. The mean nidus volume was 12.4 ± 18.4 cm^3^. Half of the AVMs (77, 50.7%) were Spetzler–Martin (SM) grades I–II. The most common VRAS score noted were VRAS = 2 (48 cases, 31.6%) and VRAS = 3 (51 cases, 33.6%), followed by VRAS = 4 (36 cases, 23.7%). The mean mRBAS score was 2.0 ± 1.9. The median interval between the embolization and SRS was 0.2 (interquartile range = 0.8) years. In terms of intraoperative details, the mean margin dose was 16.7 ± 3.1 Gy, and the maximum dose was 33.2 ± 5.0 Gy. Finally, the mean clinical follow-up duration was 6.2 ± 3.2 years, and the radiographic follow-up lasted for an average of 2.9 ± 2.3 years. After PSM, there were no statistical differences in baseline characteristics between the SRS group and Em+SRS group.

**Table 1:** Baseline characteristics of the whole cohort.

**Characteristic**	**Total**	**SRS**	**Em+SRS**	***p*-value**
	**(*n* = 152)**	**(*n* = 76)**	**(*n* = 76)**	
Female, *n* (%)	69 (45.4)	36 (47.4)	33 (43.4)	0.625
Age, mean year (SD)	29.8 (13.5)	30.6 (14.6)	29.0 (12.4)	0.454
Initial mRS score (SD)	0.9 (1.0)	0.9 (1.0)	0.9 (1.1)	0.938
Eloquent location, *n* (%)	101 (66.4)	52 (68.4)	49 (64.5)	0.606
Ruptured, *n* (%)	93 (61.2)	46 (60.5)1	47 (61.8)	0.868
Supratentorial, *n* (%)	132 (86.8)	64 (84.2)	68 (89.5)	0.337
Left hemisphere, *n* (%)	83 (54.6)	40 (52.6)	43 (56.6)	0.625
Deep venous drainage, *n* (%)	63 (41.1)	28 (36.8)	35 (46.1)	0.249
Diffuseness, *n* (%)	23 (15.1)	12 (15.8)	11 (14.5)	0.821
Deep location, *n* (%)	35 (23.0)	17 (22.4)	18 (23.7)	0.847
Aneurysm, *n* (%)	18 (11.8)	6 (7.9)	12 (15.8)	0.132
Nidus volume, ml (SD; range)	12.4 (18.4)	12.0 (18.3)	12.9 (18.7)	0.761
SM grade				0.871
I-II	77 (50.7)	38 (50.0)	39 (51.3)	
III-V	75 (49.3)	38 (50.0)	37 (48.7)	
VRAS				0.412
0–2	65 (42.8)	35 (46.1)	30 (39.5)	
3–4	87 (57.2)	41 (53.9)	46 (60.5)	
mRBAS (SD)	2.0 (1.9)	1.9 (1.9)	2.0 (1.9)	0.832
SRS margin dose, mean Gy (SD)	16.7 (3.1)	17.0 (4.0)	16.5 (1.6)	0.355
SRS maximum dose, mean Gy (SD)	33.2 (5.0)	33.6 (14.6)	29.0 (12.4)	0.356
Clinical follow-up, mean years (SD)	6.2 (3.2)	6.4 (3.5)	6.0 (2.9)	0.364
Radiological follow-up, mean years (SD)	2.9 (2.3)	3.1 (2.7)	2.8 (1.8)	0.480

*Em, embolization; mRBAS, modified radiosurgery-based AVM score; mRS, modified Rankin Scale; SD, standard deviation; SM, Spetzler–Martin; SRS. stereotactic radiosurgery; VRAS, Virginia Radiosurgery AVM Scale*.

*VRAS (Virginia Radiosurgery AVM Scale): volume 2–4 cm^3^, eloquent location, or hemorrhage = 1, volume >4 cm^3^ = 2*.

*mRBAS (modified radiosurgery-based AVM score) = 0.1 × volume (cm^3^) + 0.02 × age (years) + 0.5 × location (deep location: basal ganglia, thalamus, or brainstem = 1, else location = 0)*.

### Primary and Secondary Outcomes

**Figure 2:**
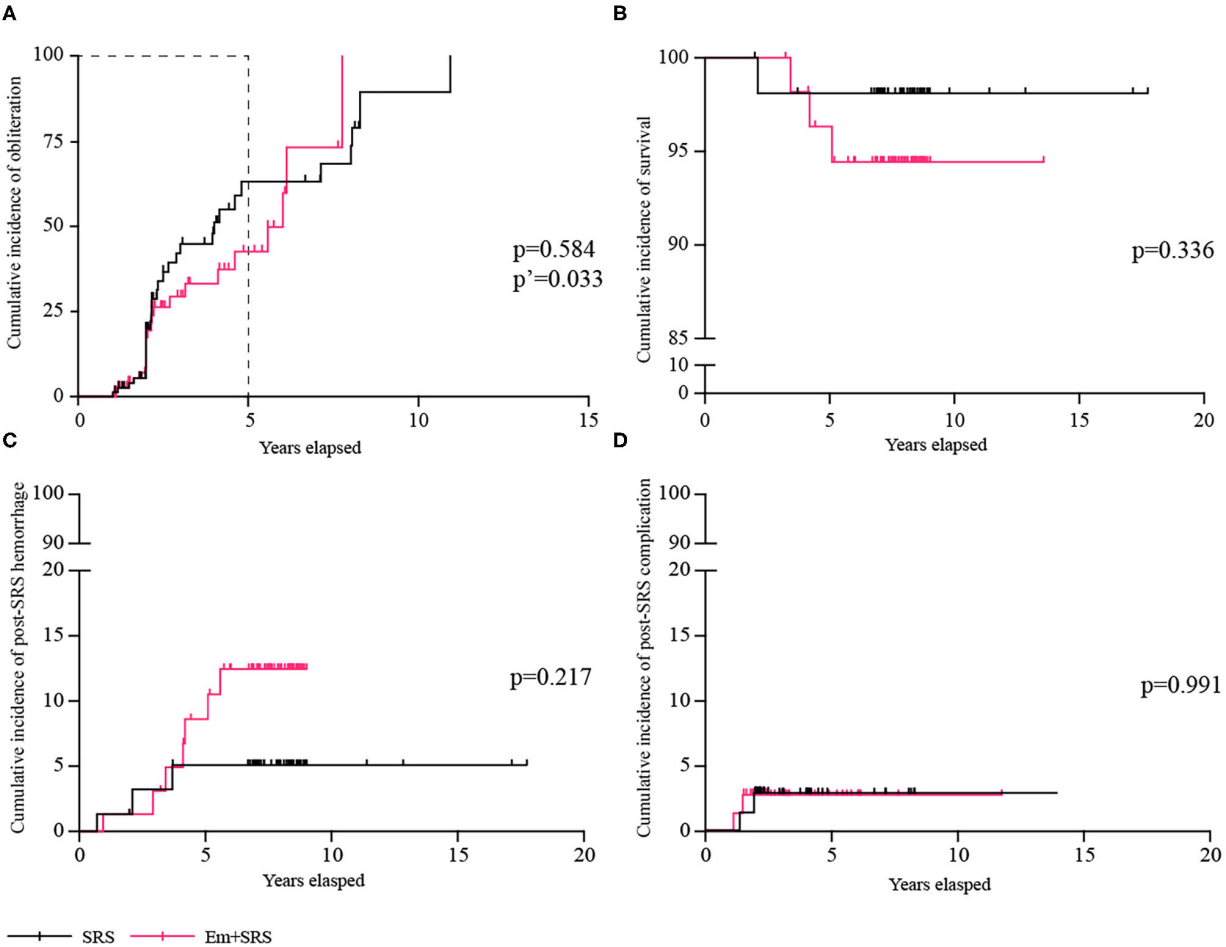
Comparisons of cumulative incidence between SRS and Em+SRS. **(A)** AVM obliteration. **(B)** Survival. **(C)** Post-SRS hemorrhage. **(D)** Post-SRS complication. AVM, arteriovenous malformation; Em+SRS, embolization + stereotactic radiosurgery. *p*′-Value refers to the result of a 5-year follow-up.

After an average of 2.9 ± 2.3 years of radiological follow-up, 58 (38.2%) patients achieved complete obliteration, and the obliteration rate was similar between SRS and Em+SRS (44.7 vs. 31.6%; OR, 1.754; 95% CI, 0.905–3.401; *p* = 0.096) (Table 2), and among these patients, 33 (56.9%) patients were confirmed by DSA (Figure 2). However, in a further analysis, the SRS group was superior to the Em+SRS group in terms of cumulative occlusion rate at a follow-up of 5 years (HR, 1.778; 95% CI, 1.017–3.110; *p* = 0.033) (Figure 2A).

**Table 2:** Primary and secondary outcomes of the whole cohort.

**Outcomes**	**Total**	**SRS**	**Em+SRS**	**OR (95% CI)**	***p*-value**
	**(*n* = 152)**	**(*n* = 76)**	**(*n* = 76)**		
**Primary outcomes**					
AVM obliteration, *n* (%)	58 (38.2)	34 (44.7)	24 (31.6)	1.754 (0.905–3.401)	0.096
**Secondary outcomes**					
Favorable functional state	143 (94.1)	73 (96.1)	70 (92.1)	2.086 (0.502–8.665)	0.312
Post-SRS hemorrhage, *n* (%)	10 (6.6)	3 (3.9)	7 (9.2)	0.405 (0.101–1.630)	0.203
All-cause mortality, *n* (%)	4 (2.6)	1 (1.3)	3 (3.9)	0.324 (0.033–3.191)	0.334
**RIC**					
Edema, *n* (%)	2 (1.3)	1 (1.3)	1 (1.3)	1.000 (0.061–16.285)	> 0.999
Cyst, *n* (%)	2 (1.3)	1 (1.3)	1 (1.3)	1.000 (0.061–16.285)	> 0.999

*AVM, arteriovenous malformation; CI, confidence interval; Em, embolization; OR, odds ratio; RIC, radiation-induced changes; SRS, stereotactic radiosurgery*.

At secondary outcomes, 143 (94.1%) patients achieved favorable functional state, and the favorable functional state was similar between SRS and Em+SRS (96.1 vs. 92.1%; OR, 2.086; 95% CI, 0.502–8.665; *p* = 0.312). Ten (6.6%) patients suffered subsequent hemorrhages after treatment, the risk of post-SRS hemorrhage was similar between these two groups (3.9 vs. 9.2%; OR, 0.405; 95% CI, 0.101–1.630; *p* = 0.203). In terms of cumulative post-SRS hemorrhage rate, it was 1.32, 5.11, and 5.11% at 2, 4, and 6 years in the SRS group, and 1.32, 6.77, and 12.48% at 2, 4, and 6 years in the Em+SRS group (HR, 0.437; 95% CI, 0.127–1.510; *p* = 0.217) (Figure 2C). Four (2.6%) patients died during clinical follow-up, one (1.3%) patient in the SRS group and three (3.9%) patients in the Em+SRS group (OR, 0.324; 95% CI, 0.033–3.191; *p* = 0.334). The cumulative all-cause mortality had no statistical differences between the two groups in the Kaplan–Meier analysis (log-rank, *p* = 0.336) (Figure 2B). In addition, edema or cyst after SRS was also similar between these two groups (log-rank, *p* = 0.991) (Figure 2D). In addition, it should be mentioned that none of the patients had serious embolic complications after upfront embolization.

### Outcomes of Ruptured Subgroup

We conducted a subgroup analysis based on hemorrhage presentation to further identify possible prognostic differences between SRS and Em+SRS. The baseline characteristics of the ruptured subgroup and unruptured subgroup are shown in the Supplementary Tables 1, 2.

**Figure 3:**
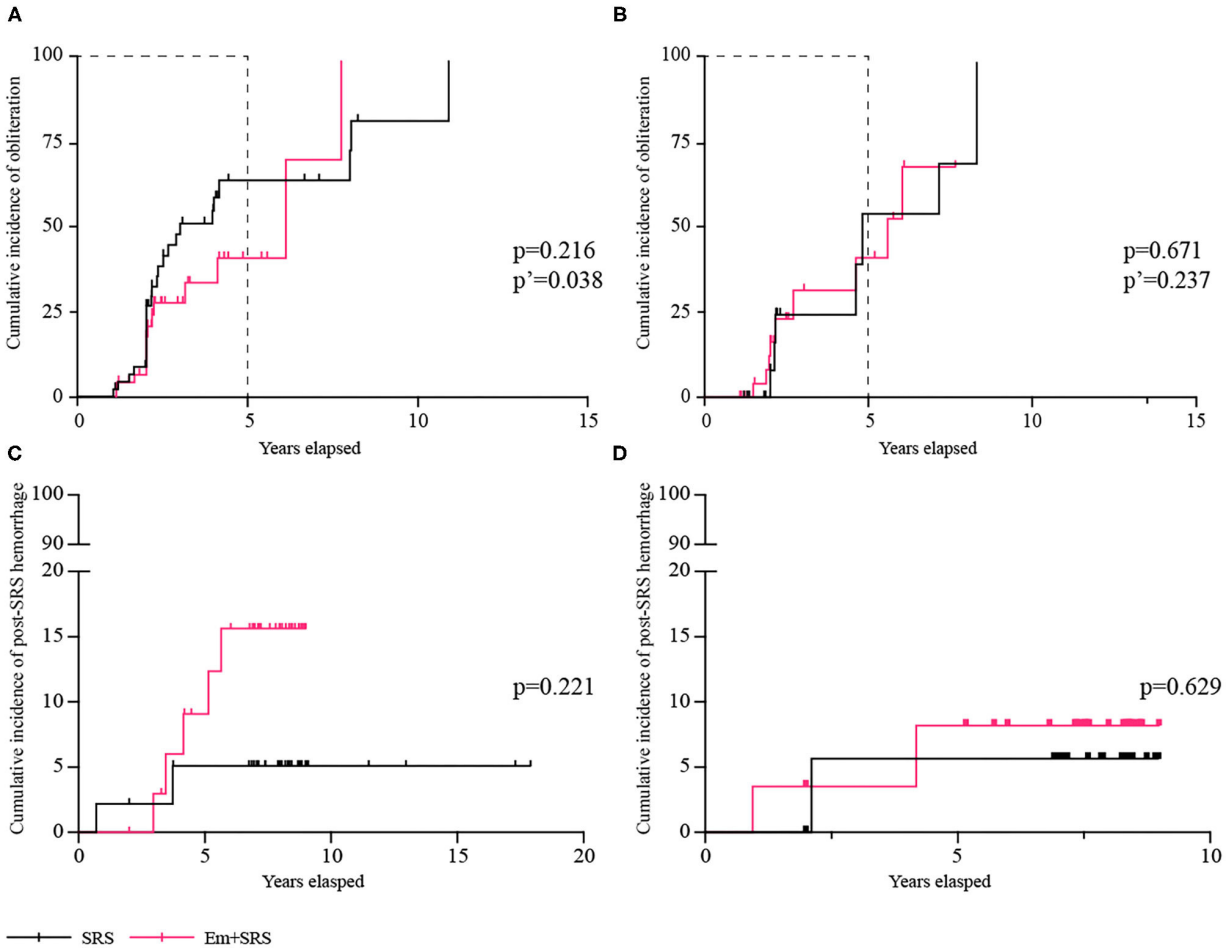
Comparisons of cumulative incidence in ruptured subgroup between SRS and Em+SRS. **(A)** AVM obliteration. **(C)** Post-SRS hemorrhage. Comparisons of cumulative incidence in the unruptured subgroup between SRS and Em+SRS: **(B)** AVM obliteration. **(D)** Post-SRS hemorrhage. AVM, arteriovenous malformation; Em+SRS, embolization + stereotactic radiosurgery. *p*′-Value refers to the result of a 5-year follow-up.

In the ruptured subgroup (*n* = 93) (Table 3), 41 (44.1%) patients achieved complete obliteration, and the SRS group could achieve higher obliteration rate than the Em+SRS group (56.5 vs. 31.9%; OR, 2.773; 95% CI, 1.190–6.464; *p* = 0.018). In the Kaplan–Meier analysis, the cumulative obliteration rate at 5 years was also higher in the SRS group than in the Em+SRS group (64.5 vs. 41.3%; HR, 2.012; 95% CI, 1.037–3.903; *p* = 0.038) (Figure 3A). In terms of secondary outcomes, including favorable functional state (95.7 vs. 89.4%; OR, 2.619; 95% CI, 0.482–14.243; *p* = 0.265), post-SRS hemorrhage (4.3 vs. 10.6%; OR, 0.382; 95% CI, 0.070–2.076; *p* = 0.265), all-cause mortality (0.0 vs. 4.3%; *p* = 0.495), edema, and cyst were all similar between these two interventional strategies. In addition, the cumulative post-SRS hemorrhage rate were similar between the two groups in the ruptured subgroup (log-rank, *p* = 0.221; Figure 3C).

**Table 3:** Primary and secondary outcomes in the ruptured subgroup.

**Outcomes**	**Total**	**SRS**	**Em+SRS**	**OR (95% CI)**	***p*-value**
	**(*n* = 93)**	**(*n* = 46)**	**(*n* = 47)**		
**Primary outcomes**					
AVM obliteration, *n* (%)	41 (44.1)	26 (56.5)	15 (31.9)	2.773 (1.190–6.464)	0.018^*^
**Secondary outcomes**					
Favorable functional state	86 (92.5)	44 (95.7)	42 (89.4)	2.619 (0.482–14.243)	0.265
Post-SRS hemorrhage, *n* (%)	7 (7.5)	2 (4.3)	5 (10.6)	0.382 (0.070–2.076)	0.265
All-cause mortality, *n* (%)	2 (2.2)	0	2 (4.3)	-	0.495
**RIC**					
Edema, *n* (%)	0	0	0	-	-
Cyst, *n* (%)	2 (2.2)	1 (2.2)	1 (2.2)	1.022 (0.062–16.845)	0.988

*AVM, arteriovenous malformation; CI, confidence interval; Em, embolization; OR, odds ratio; RIC, radiation-induced changes; SRS, stereotactic radiosurgery*.

* *Statistical significance (p < 0.05)*.

### Outcomes of Unruptured Subgroup

Among 59 patients with unruptured AVMs (Table 4), the overall obliteration rates and the cumulative obliteration rate were similar between the two groups (*p* = 0.711, *p* = 0.671, respectively) (Figure 3B). There was no significant difference between the secondary prognostic parameters of the two groups (favorable functional state, *p* = 0.981; post-SRS hemorrhage, *p* = 0.542; all-cause mortality, *p* = 0.981; edema, *p* = 0.981). In addition, the cumulative post-SRS hemorrhage rate were similar between the SRS and Em+SRS in the unruptured subgroup (log-rank, *p* = 0.629) (Figure 3D).

**Table 4:** Primary and secondary outcomes of unruptured subgroup.

**Outcomes**	**Total**	**SRS**	**Em+SRS**	**OR (95% CI)**	***p*-value**
	**(*n* = 59)**	**(*n* = 30)**	**(*n* = 29)**		
**Primary outcomes**					
AVM obliteration, *n* (%)	17 (28.8)	8 (26.7)	9 (31.0)	0.808 (0.261–2.498)	0.711
**Secondary outcomes**					
Favorable functional state	57 (96.6)	29 (96.7)	28 (96.6)	1.036 (0.062–17.377)	0.981
Post-SRS hemorrhage, *n* (%)	3 (5.1)	1 (3.3)	2 (6.9)	0.466 (0.040–5.433)	0.542
All-cause mortality, *n* (%)	2 (3.4)	1 (3.3)	1 (3.4)	0.966 (0.058–16.199)	0.981
**RIC**					
Edema, *n* (%)	2 (3.4)	1 (3.3)	1 (3.4)	0.966 (0.058–16.199)	0.981
Cyst, *n* (%)	0	0	0	-	-

*AVM, arteriovenous malformation; CI, confidence interval; Em, embolization; OR, odds ratio; RIC, radiation-induced changes; SRS, stereotactic radiosurgery*.

## Discussion

Whether pre-SRS embolization could facilitate the post-SRS obliteration for bAVMs remain controversial (11–15). We noticed that niduses in the Em+SRS group had higher SM grades and more complicated angioarchitectures when data derived from post-embolization characteristics, rather than pre-embolization lesions, were used as baseline characteristics. Therefore, we conducted a PSM analysis based on pre-embolization characteristics to compare the outcomes of SRS with and without prior embolization for bAVMs. Finally, we found that pre-SRS endovascular embolization may indeed have a negative effect on post-SRS obliteration, and the post-SRS hemorrhage and post-SRS RIC were similar between the SRS group and the Em+SRS group. In the subgroup analysis, the SRS only group have significantly higher obliteration rates than the Em+SRS group in the ruptured group, but no such phenomenon was found in the unruptured group.

SRS is considered as a reliable strategy for the treatment of small bAVMs (nidus volume <12 cm^3^ or diameter <3 cm) (16), and the obliteration rate was reported to be 56.8–80% (1, 17, 18). In this study, the long-term obliteration rate of the whole cohort was 38.2%, slightly lower than most previous studies (1), which may be due to the smaller marginal dose (16.7 ± 3.1 Gy) and larger nidus volume (12.4 ± 18.4 cm^3^) in our study. A combination of embolization and SRS is frequently used to treat large bAVMs on the basis of the assumption that prior embolization may facilitate post-SRS obliteration of the residual lesion by reducing the nidus volume and slowing the nidus blood flow (3, 14). However, many recent studies revealed that prior partial embolization may have a negative effect on post-SRS obliteration (1). Russell et al. conducted a meta-analysis to find that the combination strategy is associated with lower obliteration rates than SRS alone (48.4 vs. 62.7%) (1). In this study, we also found that the combination strategy has lower obliteration rate than that of the SRS alone strategy (31.6 vs. 44.7%, no statistical difference). However, it should be noted that the obliteration rate of the combined strategy was significantly lower than that of the SRS alone strategy alone at the end of the 5-year follow-up post-operatively (*p* = 0.033). Several hypotheses have been proposed to explain why partial nidus embolization could decrease obliteration rates after SRS. Partial nidus embolization may fragment the nidus into scattered sections, thus, transforming a compact nidus into a diffuse one, and consequently increasing the difficulty of target delineation (19, 20). Moreover, embolic agents may suppress the obliteration rates by several mechanisms, including dose reduction by radiation absorption or scattering (21), obscuration of the residual nidus on post-embolization neuroimaging, and recanalization or pseudo-occlusion (11). Furthermore, embolization may stimulate and promote angiogenesis of bAVMs, consequently increasing the radioresistance of residual nidus (22, 23). Our team believes that the fragmentation caused by partial embolization may be the main causes leading to the decrease in obliteration rate, which makes it impossible to accurately locate the nidus boundary when making the irradiation plan. However, we also noted that the mRBAS of our cohort was 2.0 ± 1.9. Based on our previous study (18), the patients with mRBAS >1.5 seem to be inclined to be more suitable for SRS only instead of the combination strategy.

In this study, we found no significant difference in post-SRS hemorrhage between the two strategies. Pre-SRS embolization was usually used to target angiographic features with a high risk of bleeding, such as comorbid aneurysms and arteriovenous fistulas (13). However, some previous studies reported that the Em+SRS strategy may have higher post-SRS bleeding rate (1), the potential mechanism of which may be the increased vascular stress in residual lesions after partial embolization, or targeted embolization of high-risk bleeding factors cannot effectively reduce the risk of rupture (24, 25). The edema and cyst were similar between the two strategies, and the overall rate of edema and cyst in our study was relatively lower than in previous studies. Several studies suggested that RIC was associated with a higher margin dose (26), and partial embolization could reduce the risk of RIC (27). The margin dose in our study was generally low (16.7 ± 3.1 Gy), which may be the cause of the lower incidence of edema and cyst.

Several previous studies indicated lower obliteration rates for unruptured bAVMs compared with ruptured AVMs after SRS (28, 29). The investigators postulated a possible synergism between radiation and hemorrhage for AVM obliteration *via* mechanisms of endothelial damage, myofibroblast proliferation, and progressive endoluminal occlusion and thrombosis (30, 31). Nevertheless, previous studies did not explore the obliteration rate of the SRS strategy and Em+SRS strategy in the ruptured and unruptured subgroups. In this study, the SRS group was found to have significantly higher obliteration rates than the Em+SRS group in the ruptured cohort. While no such phenomenon was found in the unruptured cohort, we surmised that embolization may disturb the synergism and lead to lower obliteration rates.

Therefore, it is more reasonable to adopt SRS alone for ruptured bAVMs, while for unruptured bAVMs, both strategies are acceptable.

However, is the combination strategy useless? Of course not. Hemodynamics is thought to be closely related to the biological behavior and development of bAVM (32–34), and it is traditionally believed that fast and large blood flow is not conducive to nidus obliteration after SRS (17). Hu et al. suggested that stagnant venous outflow predicts bAVM obliteration after Gamma knife radiosurgery by quantitative DSA (35), and Rivera et al. proposed that partial embolization could prolong the time to peak values at the arterial feeder, drainage vein, and venous sinus (36). Therefore, choosing a reasonable target endovascular embolization strategy that is hemodynamically beneficial to the nidus obliteration may be the next research direction of the combination strategy.

### Limitation

Despite our best efforts to improve the design defects of previous researches by adjusting for baseline differences and selection biases using PSM. There were still several limitations: the main limitations were its retrospective nature. Some patients cannot maintain strict and regular imaging follow-up, so we cannot know the exact time of nidus occlusion in these patients. Another limitation is that not all patients had DSA to confirm obliteration. In order to reduce the deviation caused by this limitation, we commissioned two senior neuroradiologists to evaluate the last radiographic follow-up independently, and a third more senior professor-level experts will reevaluate if the result is controversial.

## Conclusion

Although there was no significant difference in the overall obliteration rate between the two strategies, pre-SRS endovascular embolization may have a negative effect on post-SRS obliteration and did not negatively affect post-SRS hemorrhage and complications for bAVMs. In the subgroup analysis, the obliteration rates of SRS only strategy was significantly higher than that of the Em+SRS strategy in the ruptured cohort, while no such phenomenon was found in the unruptured cohort.

## Data Availability Statement

The data analyzed in this study is subject to the following licenses/restrictions: Data will be shared with qualified investigators upon request. Requests to access these datasets should be directed to Yuanli Zhao, zhaoyuanli@126.com.

## Ethics Statement

The studies involving human participants were reviewed and approved by the Ethics Committee of Beijing Tiantan Hospital, China and the ethics number was KY 2020-003-01. Written informed consent to participate in this study was provided by the participants' legal guardian/next of kin.

## Author Contributions

DY and YZ conceptualized and designed the study. YL, SS, and AL were the operators. ZL, HZ, KY, RL, XM, HJ, and DG acquired the data. DY, YC, and HH analyzed and interpreted the data. DY wrote and prepared the original draft. XC, SS, and YZ wrote, reviewed, and edited the manuscript. YZ supervised the study. All authors contributed to the article and approved the submitted version.

## Funding

This study was funded by the National Natural Science Foundation of China (H0906 81571110 and 82071302 to YZ).

## Conflict of Interest

The authors declare that the research was conducted in the absence of any commercial or financial relationships that could be construed as a potential conflict of interest.

## Publisher's Note

All claims expressed in this article are solely those of the authors and do not necessarily represent those of their affiliated organizations, or those of the publisher, the editors and the reviewers. Any product that may be evaluated in this article, or claim that may be made by its manufacturer, is not guaranteed or endorsed by the publisher.

## Supplementary Material

Supplementary Figure 1 Standardized differences of baseline characteristics between brain AVM patients who received stereotactic radiosurgery with or without prior embolization before after matching. AVM, arteriovenous malformation; VRAS, Virginia Radiosurgery AVM Scale; mRBAS, modified radiosurgery-based AVM score; Dashed vertical lines indicate standardized differences of 0.2.

Supplementary Figure 2 Distribution of Radiological follow-up methods between different primary outcomes. MRI, magnetic resonance imaging; DSA, digital subtraction angiography.

Supplementary Table 1 Baseline characteristics of prior hemorrhage subgroup.

Supplementary Table 2 Baseline characteristics of unruptured subgroup.
