# Supplementary figures and images for: Stereotactic Radiosurgery With vs. Without Prior Embolization for Brain Arteriovenous Malformations: A Propensity Score Matching Analysis
Source: Front Neurol. 2021 Oct 12;12:752164. doi: 10.3389/fneur.2021.752164 (PMC8545857; doi:10.3389/fneur.2021.752164)

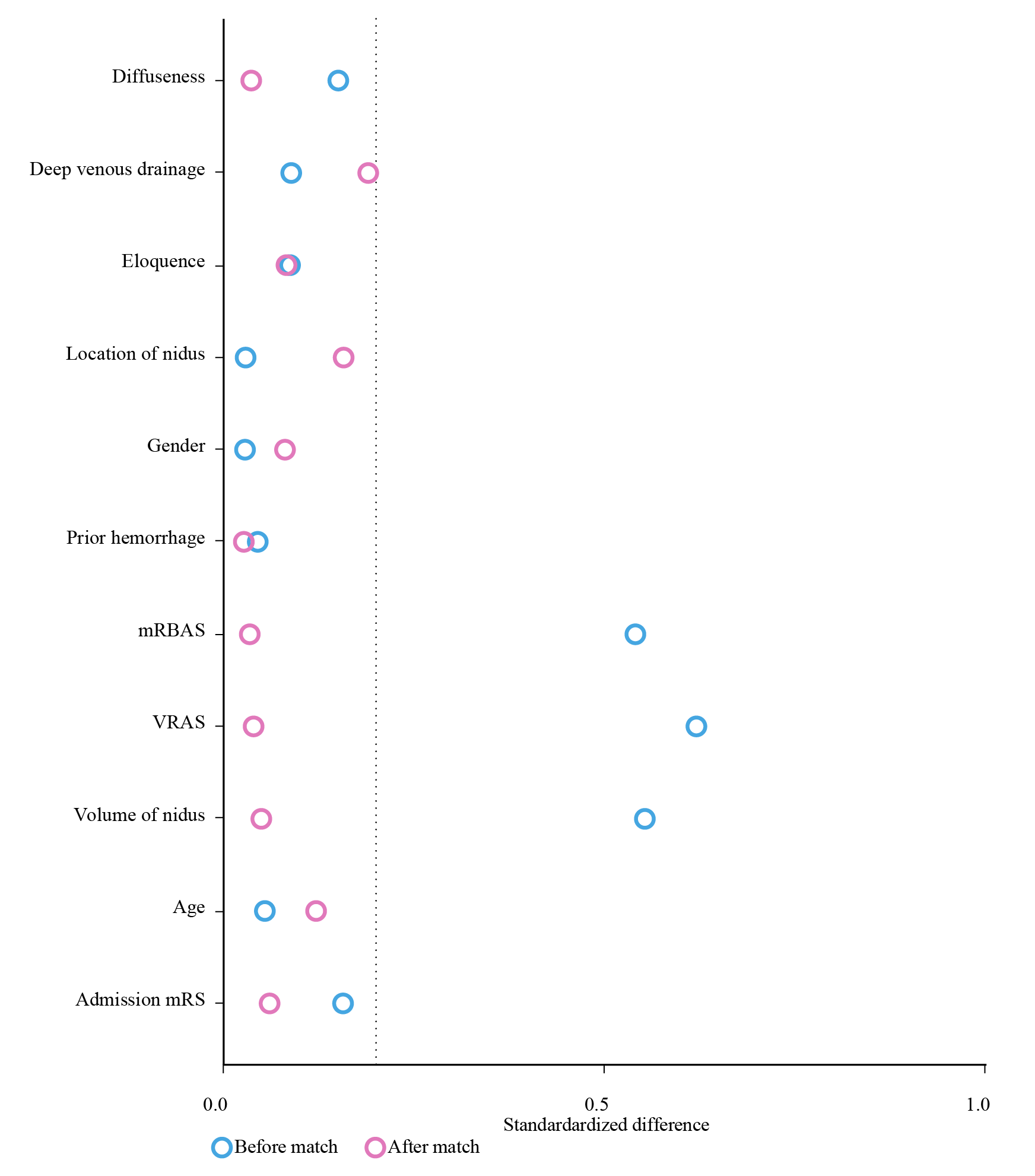

Supplement: Supplementary Figure 1 — Standardized differences of baseline characteristics between brain AVM patients who received stereotactic radiosurgery with or without prior embolization before after matching. AVM, arteriovenous malformation; VRAS, Virginia Radiosurgery AVM Scale; mRBAS, modified radiosurgery-based AVM score; Dashed vertical lines indicate standardized differences of 0.2. [file Image_1.TIF]

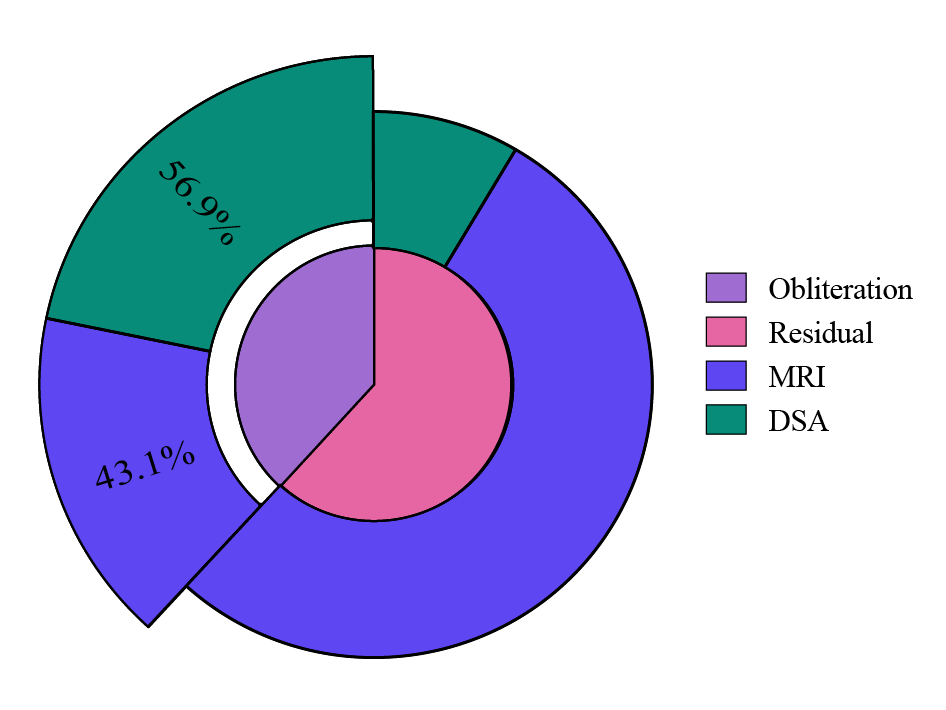

Supplement: Supplementary Figure 2 — Distribution of Radiological follow-up methods between different primary outcomes. MRI, magnetic resonance imaging; DSA, digital subtraction angiography. [file Image_2.TIF]
