# Supplementary material for: Stereotactic Radiosurgery With vs. Without Prior Embolization for Brain Arteriovenous Malformations: A Propensity Score Matching Analysis
Source: Front Neurol. 2021 Oct 12;12:752164. doi: 10.3389/fneur.2021.752164 (PMC8545857; doi:10.3389/fneur.2021.752164)
Supplement: Supplementary Table 1 — Baseline characteristics of prior hemorrhage subgroup. [file Table_1.docx]

Table I. Baseline characteristics of prior hemorrhage subgroup

| Characteristic | SRS  (n=46) | Em+SRS  (n=47) | p value |
| --- | --- | --- | --- |
| Female, n (%) | 21(45.7) | 21(44.7) | 0.925 |
| Age, mean yr (SD) | 26.84(12.93) | 29.13(11.85) | 0.375 |
| Initial mRS score (SD) | 1.11(1.14) | 1.04(1.23) | 0.789 |
| Eloquent location, n (%) | 33(71.7) | 33(70.2) | 0.871 |
| Supratentorial, n (%) | 38(82.6) | 41(87.2) | 0.533 |
| Left hemisphere, n (%) | 27(58.7) | 25(53.2) | 0.593 |
| Deep venous drainage, n (%) | 20(43.5) | 24(51.1) | 0.464 |
| diffuseness, n (%) | 10(21.7) | 8(17.0) | 0.565 |
| Deep location, n (%) | 14(30.4) | 15(31.9) | 0.878 |
| Aneurysm, n (%) | 4(8.7) | 7(14.9) | 0.355 |
| Nidus volume, ml (SD; range) | 12.54(22.38) | 9.35(11.39) | 0.387 |
| SM grade  I-II  III -V | 22(47.8)  24(52.2) | 22(46.8)  25(53.2) | 0.922 |
| VRAS  0-2  3-4 | 20(43.5）  26(56.5) | 15(31.9)  32(68.1) | 0.250 |
| mRBAS (SD) | 1.94(2.28) | 1.68(1.21) | 0.483 |
| SRS margin dose, mean Gy (SD; range) | 16.78(1.52) | 16.87(1.47) | 0.773 |
| SRS maximum dose, mean Gy (SD; range) | 33.32(3.10) | 33.55(2.88) | 0.712 |
| Clinical follow-up, mean years (SD) | 7.15(3.70) | 5.86(3.04) | 0.069 |
| Radiological follow-up, mean years (SD) | 3.33(3.09) | 2.79(1.79) | 0.306 |

Em= Embolization; mRBAS= modified Radiosurgery-Based AVM Score; mRS= modified Rakin Scale; SD= Standard Deviation; SM= Spetzler Martin; SRS= Stereotactic Radiosurgery; VRAS= Virginia Radiosurgery AVM Scale

VRAS (Virginia Radiosurgery AVM Scale): volume 2-4 cm^3^, eloquent location, or hemorrhage = 1, volume > 4 cm^3^ = 2.

mRBAS (modified radiosurgery-based AVM score) = 0.1×volume (cm^3^) + 0.02×age (years) + 0.5×location (deep location: basal ganglia, thalamus, or brainstem = 1, else location = 0)

Table II. Baseline characteristics of unruptured subgroup

| Characteristic | SRS  (n=30) | Em+SRS  (n=29) | p value |
| --- | --- | --- | --- |
| Female, n (%) | 15(50.0) | 12(41.4) | 0.506 |
| Age, mean yr (SD) | 36.45(15.17) | 28.75(13.42) | 0.044* |
| Initial mRS score (SD) | 0.67(0.48) | 0.72(0.88) | 0.759 |
| Eloquent location, n (%) | 19(63.3) | 16(55.2) | 0.524 |
| Supratentorial, n (%) | 26(86.7) | 27(93.1) | 0.533 |
| Left hemisphere, n (%) | 13(43.3) | 18(62.1) | 0.150 |
| Deep venous drainage, n (%) | 8(26.7) | 11(37.9) | 0.355 |
| diffuseness, n (%) | 2(6.7) | 3(10.3) | 0.968 |
| Deep location, n (%) | 3(10.0) | 3(10.3) | 1.000 |
| Aneurysm, n (%) | 2(6.7) | 5(17.2) | 0.394 |
| Nidus volume, ml (SD; range) | 11.13(9.19) | 18.65(25.86) | 0.148 |
| SM grade  I-II  III -V | 16(53.3)  14(46.7) | 17(58.6)  12(41.4) | 0.683 |
| VRAS  0-2  3-4 | 15(50.0)  15(50.0) | 15(51.7)  14(48.3) | 0.895 |
| mRBAS (SD) | 1.89(0.93) | 2.49(2.63) | 0.255 |
| SRS margin dose, mean Gy (SD; range) | 17.23(6.19) | 15.90(1.59) | 0.264 |
| SRS maximum dose, mean Gy (SD; range) | 34.05(9.54) | 31.72(3.23) | 0.217 |
| Clinical follow-up, mean years (SD) | 5.35(3.03) | 6.13(2.73) | 0.306 |
| Radiological follow-up, mean years (SD) | 2.68(1.94) | 2.84(1.85) | 0.741 |

Em= Embolization; mRBAS= modified Radiosurgery-Based AVM Score; mRS= modified Rakin Scale; SD= Standard Deviation; SM= Spetzler Martin; SRS= Stereotactic Radiosurgery; VRAS= Virginia Radiosurgery AVM Scale

VRAS (Virginia Radiosurgery AVM Scale): volume 2-4 cm^3^, eloquent location, or hemorrhage = 1, volume > 4 cm^3^ = 2.

mRBAS (modified radiosurgery-based AVM score) = 0.1×volume (cm^3^) + 0.02×age (years) + 0.5×location (deep location: basal ganglia, thalamus, or brainstem = 1, else location = 0)

^*^Statistical significance (p<0.05)
